# Atypical Kawasaki Disease Presentation in a Previously Healthy Infant: A Diagnostic Challenge

**DOI:** 10.7759/cureus.46748

**Published:** 2023-10-09

**Authors:** Ali Aizad Raza, Warisha Khan, Arshan A Khan, Syed Kanza Mahrukh, Krishnan Balasubramanian

**Affiliations:** 1 Pediatrics, Tunbridge Wells Hospital, Maidstone and Tunbridge Wells NHS Trust, Tunbridge Wells, GBR; 2 Internal Medicine, Faisalabad Medical University, Faisalabad, PAK; 3 Internal Medicine, Ascension St. John Hospital, Grosse Pointe Woods, USA; 4 Internal Medicine, Sir Ganga Ram Hospital, Lahore, PAK

**Keywords:** kawasaki disease in atypical age group, kawasaki disease diagnostic challenges, kawasaki disease treatment, atypical kawasaki disease, kawasaki disease (kd)

## Abstract

Kawasaki disease (KD) is an acute systemic vasculitis primarily affecting children, characterized by fever and multisystem involvement. We present a compelling case of KD in a previously healthy 13-week-old infant who presented with fever, irritability, reduced feeding, and the subsequent development of classical mucocutaneous manifestations, including bilateral non-purulent conjunctivitis, cracked lips, and an erythematous rash. Laboratory findings revealed elevated inflammatory markers, thrombocytosis, and neutrophilic leukocytosis, consistent with the diagnosis. The patient was started on intravenous immunoglobulins (IVIG) at a dose of 2g/kg, IV methylprednisolone, and a high dose of aspirin. The infant was eventually transferred to a tertiary care hospital for comprehensive management. The case is intriguing due to its presentation in an atypical age group. Prompt recognition and management of KD are crucial to prevent the development of coronary artery abnormalities. This case underscores the importance of considering KD in the differential diagnosis of infants with fever and unusual clinical presentations, even in the absence of typical cardiac involvement. Early identification and appropriate treatment are essential to prevent potential complications and improve outcomes.

## Introduction

Kawasaki disease (KD), initially described by Dr. Tomisaku Kawasaki in 1967, is a rare but significant pediatric vasculitis primarily affecting infants and children. This illness is characterized by a constellation of clinical features, including prolonged fever, mucocutaneous manifestations, lymphadenopathy, and systemic inflammation. Although KD is relatively uncommon, it holds the dubious distinction of being the leading cause of acquired heart disease among children in developed nations, particularly in Japan and the United States [1].

Central to KD's clinical significance is its propensity to involve the coronary arteries, potentially leading to coronary artery aneurysms and other severe cardiovascular complications if not expeditiously identified and managed. As such, the early recognition and appropriate treatment of KD are paramount in preventing adverse long-term outcomes [2]. Here, we present a unique case of KD in a 13-week-old previously healthy infant.

## Case presentation

A previously healthy 13-week-old infant, with a weight of 6.2 kg, who had received immunizations six days prior, was brought to our facility with a one-day history of fever, irritability, and markedly reduced feeding, amounting to less than half of their usual intake. Initial vital signs indicated a fever of 38.2°C, a heart rate of 161 beats per minute, oxygen saturation at 99%, and a respiratory rate of 38 breaths per minute. The infant exhibited pronounced irritability, restlessness, and mottling, while systemic examination initially revealed no remarkable findings. Laboratory investigations, however, uncovered elevated inflammatory markers: a C-reactive protein (CRP) level of 48 mg/L, a white cell count (WCC) of 17 x 10^9^/L, neutrophilic leukocytosis with a neutrophil count of 10.4 x 10^9^/L, and thrombocytosis with a platelet count of 452 x 10^9^/L. The blood culture, stool culture, urine culture, and extended viral panel were negative. Liver ultrasound and liver function test were unremarkable. A blood film analysis revealed neutrophil left shift and toxic granulation, suggesting an underlying inflammatory process. In response to the suspicion of infection, the infant was promptly screened and treated for sepsis, receiving an intravenous (IV) saline bolus (10 ml/kg) and commencing IV ceftriaxone (80mg/kg) therapy. Subsequently, he developed loose stools and mild respiratory distress with recessions, grunting, and head bobbing, prompting the initiation of Vapotherm (Vapotherm Inc., Exeter, New Hampshire, United States)/Optiflow (Fisher & Paykel Healthcare Corporation Limited, Auckland, New Zealand) therapy at 21%. Despite these interventions, fever persisted, and clinical improvement remained elusive. A lumbar puncture was performed to rule out meningitis, but cerebrospinal fluid (CSF) analysis returned normal.

**Figure 1:**
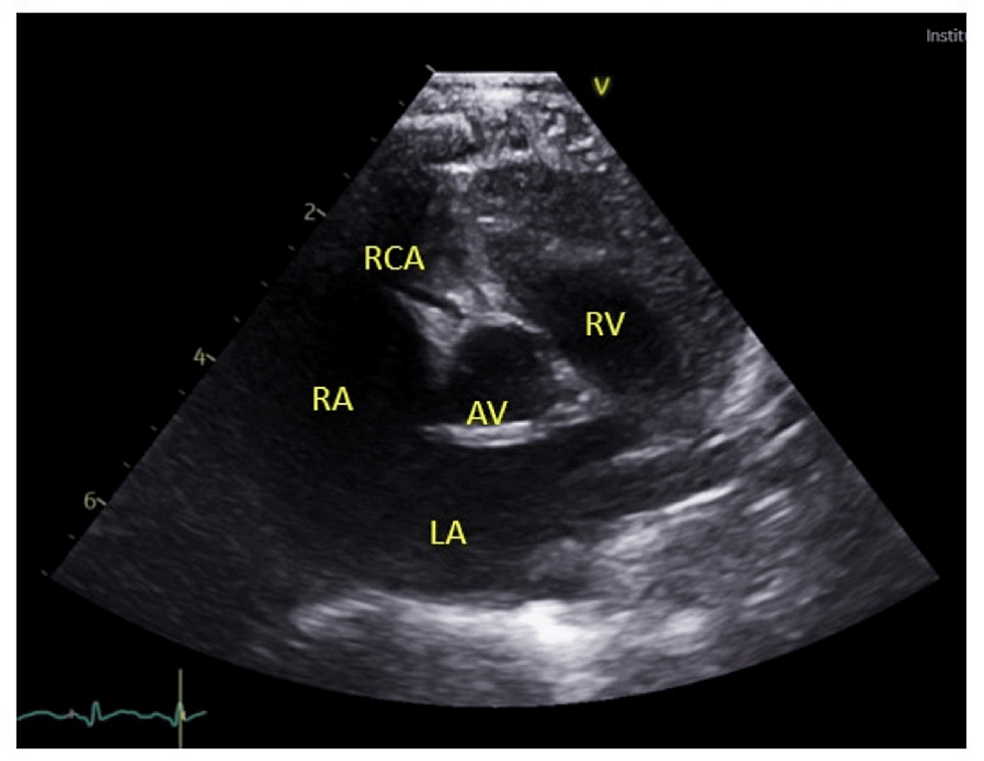
An echocardiogram (ECHO) showing normal-size RCA RCA: right coronary artery, RA: right atrium, LA: left atrium, RV: right ventricle, AV: aortic valve

**Figure 2:**
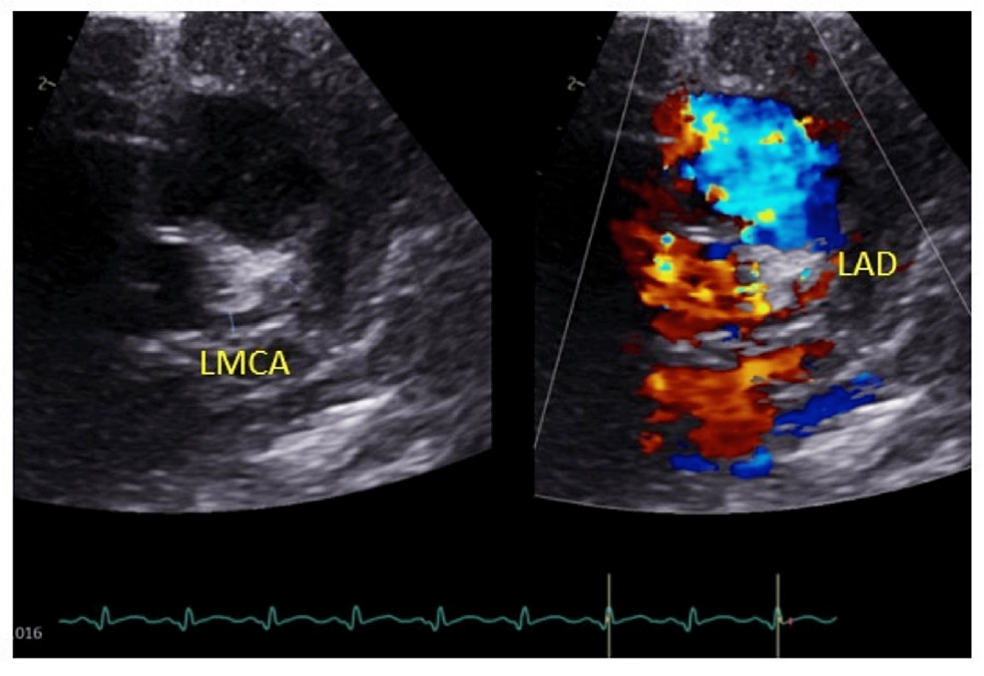
An echocardiogram (ECHO) showing prominent LMCA LMCA: left main coronary artery, LAD: left anterior descending artery

On the second day of admission, the infant developed bilateral non-purulent conjunctivitis, cracked lips, and an erythematous maculopapular blanching rash involving the face and body. Systemic examination revealed an unremarkable cardiovascular system, with S1 and S2 heart sounds and no murmurs. Respiratory examination on Vapotherm/Optiflow showed mild work of breathing with no added sounds, and auscultation revealed good bilateral air entry. Abdominal examination demonstrated a soft abdomen without visceromegaly, while the central nervous system (CNS) examination revealed normal tone and reflexes. The persistent fever, coupled with the emergence of cutaneous and mucous membrane manifestations, led to a suspicion of KD. An echocardiogram (ECHO) was performed, revealing prominent coronary arteries (Figures 1-2), further prompting discussions with the pediatric infectious disease and cardiology teams at the tertiary care facility. Following consultation with the pediatric infectious disease team, the decision was made to treat the patient as a case of KD. The patient was started on IV immunoglobulins (IVIG) at a dose of 2g/kg and a high dose of aspirin (75 mg QID). The infant was eventually transferred to a tertiary care hospital for comprehensive management.

The patient didn't respond to IVIG, and the decision was made to start the patient on IV methylprednisolone at the tertiary care facility and to have the patient complete a three-day course, followed by an oral steroid-weaning regimen. High-dose aspirin therapy was administered initially, transitioning to low-dose aspirin as the fever subsided after three days. Subsequently, the infant's clinical condition improved, including gradual progress in oral feeds. A serial ECHO demonstrated normal cardiac function with no structural abnormalities. Consequently, the infant was discharged and prescribed an oral prednisolone-weaning regimen, with instructions to continue low-dose aspirin for six weeks alongside omeprazole. Vaccinations were deferred until three months following IVIG treatment. Post-discharge follow-up included a two-week appointment at the KD clinic and subsequent reviews in the General Pediatrics clinic locally.

## Discussion

KD is a rare but serious systemic vasculitis primarily affecting children, with potentially devastating cardiovascular complications if left untreated. Although the exact cause of KD remains unknown, it is believed to involve an abnormal immune response, possibly triggered by an infectious agent, in genetically predisposed individuals [1]. The American Heart Association diagnostic criteria for KD are well-established and include fever persisting for five or more days, along with at least four of the following clinical features: bilateral non-purulent conjunctival injection, changes in the oral mucous membranes (e.g., "strawberry tongue"), cervical lymphadenopathy, rash, and changes in the extremities (e.g., swelling, erythema) [2]. These criteria aid in the early identification of KD, facilitating prompt treatment to mitigate the risk of coronary artery abnormalities.

KD exhibits epidemiological variations, with a higher incidence in Asian populations, particularly in Japan, where it was initially described by Dr. Tomisaku Kawasaki in 1967 [3]. Furthermore, KD has a seasonal predilection, with a higher incidence during late winter and early spring [4]. The clinical presentation of KD can be challenging, as it encompasses a wide array of symptoms. Fever is the cardinal feature, often lasting for several days before other manifestations become apparent. The characteristic mucocutaneous findings include conjunctival injection, red and cracked lips, a "strawberry tongue," and an erythematous rash. The rash can evolve, eventually becoming generalized and leading to desquamation, particularly of the fingertips [5].

In addition to clinical evaluation, laboratory investigations play a pivotal role in supporting the diagnosis of KD. Patients typically exhibit elevated inflammatory markers, such as CRP and erythrocyte sedimentation rate (ESR). Thrombocytosis and neutrophilic leukocytosis are common hematological findings. A blood film analysis may reveal neutrophil left shift and toxic granulation, indicating an underlying inflammatory process [6]. Imaging studies, particularly echocardiography, are crucial in assessing coronary artery involvement, as KD's most serious complication is coronary artery aneurysms. Early treatment with IVIG and aspirin is key to reducing the risk of coronary artery abnormalities [7]. Serial echocardiograms are often performed during the acute phase and in the following months to monitor coronary artery size and function [8]. In addition to coronary artery complications, KD can lead to other cardiovascular sequelae, including myocarditis, pericarditis, and valvulitis [9]. However, the prognosis for KD is generally favorable when diagnosed and treated promptly. Mortality rates have significantly decreased with IVIG therapy, but the risk of coronary artery abnormalities remains a concern. Adequate follow-up and cardiology assessments are essential to monitor coronary artery health and overall cardiovascular status [10].

In this case, we present an exceptional occurrence of KD in a 13-week-old infant, which is significantly younger than the typical age of onset. KD primarily affects children, with the majority of cases diagnosed in children between one and three years of age [3-4]. However, the age of this patient highlights the importance of recognizing that KD can manifest in even younger infants, necessitating a heightened index of suspicion in the evaluation of febrile infants, particularly those with atypical clinical presentations.

Our case presentation aligns with several key features of KD, including fever persisting for more than five days, cracked lips, and an erythematous maculopapular rash. The patient's age at onset, along with the absence of some key clinical features such as conjunctival injection, swollen hands and feet, strawberry tongue, and mucous membrane changes, made the diagnosis challenging and emphasized the need for vigilance in considering KD even in the absence of all the typical features of KD.

## Conclusions

KD remains a diagnostic challenge, particularly when it presents with atypical clinical features in infants. The atypical age of onset in this case serves as a reminder to healthcare providers that KD, although rare, should remain within the differential diagnosis even in very young infants. Early recognition and intervention are critical in averting potential serious complications, particularly coronary artery abnormalities. The rapid recognition and management of KD are pivotal to preventing potentially severe cardiovascular complications such as coronary artery aneurysms, depressed myocardial contractility and heart failure, myocardial infarction, and arrhythmias. Early identification and appropriate treatment are essential for improving outcomes in affected children.
